# A fRAmework of the DetermInants of Arts aNd Cultural Engagement (RADIANCE): integrated insights from ecological, behavioural and complex adaptive systems theories

**DOI:** 10.12688/wellcomeopenres.21625.1

**Published:** 2024-07-05

**Authors:** Daisy Fancourt, Katey Warran

**Affiliations:** 1 Department of Behavioural Science and Health, University College London, London, England, UK; 2 School of Health in Social Science, University of Edinburgh, Edinburgh, UK

**Keywords:** arts, equity, socio-economic, inequalities, barriers, determinants, public health

## Abstract

**Background:**

Arts and cultural engagement (ACEng) is ubiquitous across every human culture since palaeolithic times, but in contemporary society, ACEng is unevenly distributed, demographically, socio-economically, geographically and politically. But what are the “determinants” of ACEng (i.e., the facilitators or barriers to people’s engagement) and how can they be optimised? Despite a large body of theory and evidence on individual determinants, this work has largely occurred in disciplinary silos, which has led variously to contrasting discourses and approaches, criticism, and inconsistent findings. What we lack is a rigorous comprehensive understanding of these determinants (both those already theorised and those that have been little recognised as determinants to date) that goes beyond descriptively showing inequalities, instead explaining why these inequalities exist and how they can be overcome. This paper explores the currently recognised determinants of ACEng, and existing theoretical approaches to these determinants.

**Methods:**

Drawing on the theoretical bases of ecological systems theory, ecosocial theory and complex adaptive systems science, we conducted a review and iterative theorising process.

**Results:**

We propose a new theoretical framework of the determinants of arts and cultural engagement (RADIANCE) developed through cross-disciplinary literature reviewing, domain mapping, and consensus building.

**Conclusions:**

Overall, we identified 35 different factors that can act as determinants of ACEng across micro, meso, exo, macro and chrono levels. We broadly categorised these as social (i.e. a primary feature being the interaction of people), tangible (i.e. a primary feature involving physical assets or resources or the production of physical assets), and intangible (i.e. constructs that do not have a primary physical basis but instead have a virtual or imaginary basis). The relevance and implications of this framework for broader research, policy, and practice and case studies of it in use are presented.

## Plain language summary

All cultures across history engage in arts and cultural activities. But in modern-day society, arts engagement is unevenly patterned. Why is this and what are the factors that prevent people from engaging equitably? Much research on this topic has, to-date, happened in silos. We lack an overall theoretical framework for the barriers and enablers of arts engagement. In this article, we propose a new framework (RADIANCE) that we have developed through literature reviewing and engagement with disciplinary experts. RADIANCE proposes 35 factors that act as determinants of arts engagement at individual, community and societal levels. We define these factors and explain their interconnections as well as exploring the implications of RADIANCE for research, policy and practice.

## Introduction

Arts and cultural engagement (ACEng) is ubiquitous across every human culture since at least palaeolithic times ( Moro Abadía & González Morales, 2013). A range of literature has shown that infants display innate musicality, and response to artistic stimuli and artistic engagement is embedded within and across human rituals throughout the life course ( Dissanayake, 2008). Evolutionary anthropologists and broader theorists have even debated adaptive benefits of ACEng, including playing roles in sexual selection, social bonding, neurocognitive development, and communication ( Miller, 2001; Tooby & Cosmides, 2001). The important role of ACEng individually and collectively continues to be acknowledged in contemporary society, with a burgeoning research literature over the past few decades demonstrating the social and cultural value of ACEng, including for education, criminal justice, society, health, and wellbeing ( Crossick & Kaszynska, 2016).

However, in contemporary society, ACEng is unevenly distributed, demographically, socio-economically, geographically and politically ( Falk & Katz-Gerro, 2016; Rius-Ulldemolins *et al.*, 2019; Srakar *et al.*, 2018). Preliminary evidence suggests that some inequalities in ACEng (e.g. socio-economic inequalities) may be expanding over time ( Bone *et al.*, 2021), while others are more pronounced in countries with greater socio-economic inequalities ( Falk & Katz-Gerro, 2016). This inequality in engagement goes against the Universal Declaration of Human Rights (UDHR), which established in 1948 the right to ‘participate in the cultural life of the community’ and ‘to enjoy the arts’ ( United Nations General Assembly, 1949). The majority of UN member states have formally accepted these responsibilities in international treaties associated with the UDHR and it has been given legal status in international law by two treaties ( Veal, 2023). But while countries’ records on other human rights are held to public scrutiny, the same scrutiny is not applied to ACEng. Further, in light of copious research documenting the societal benefits of ACEng ( Crossick & Kaszynska, 2016; Fancourt & Finn, 2019; Zbranca *et al.*, 2022), this inequality in access could be exacerbating social inequalities, meaning it should be considered a concern for domains including public health, education, and other public services.

This uneven participation in ACEng is likely determined by our societies rather than by our biology, particularly in light of evolutionary-anthropological research on the historical ubiquitous nature of arts practices globally. But it raises a crucial question: what are the “determinants” of ACEng (i.e., the factors that act as facilitators or barriers to people’s engagement) and how can they be optimised? Such a question is vital to explore to work towards ensuring equity in ACEng, ensuring that all those who wish to access and engage in the arts can, and supporting the development of policies and strategies at a government level. Thus, this paper will first explore the currently recognised determinants of ACEng, and existing theoretical approaches to these determinants. Then we will consider limitations of current theories, and how the application of alternative theoretical lenses could advance our understanding. It will then propose a new theoretical framework of the determinants of arts and cultural engagement (RADIANCE) developed through cross-disciplinary literature reviewing, domain mapping, and consensus building. The relevance of this framework for broader research, policy, and practice will be discussed.

### Determinants of arts and cultural engagement

**Figure 1.**
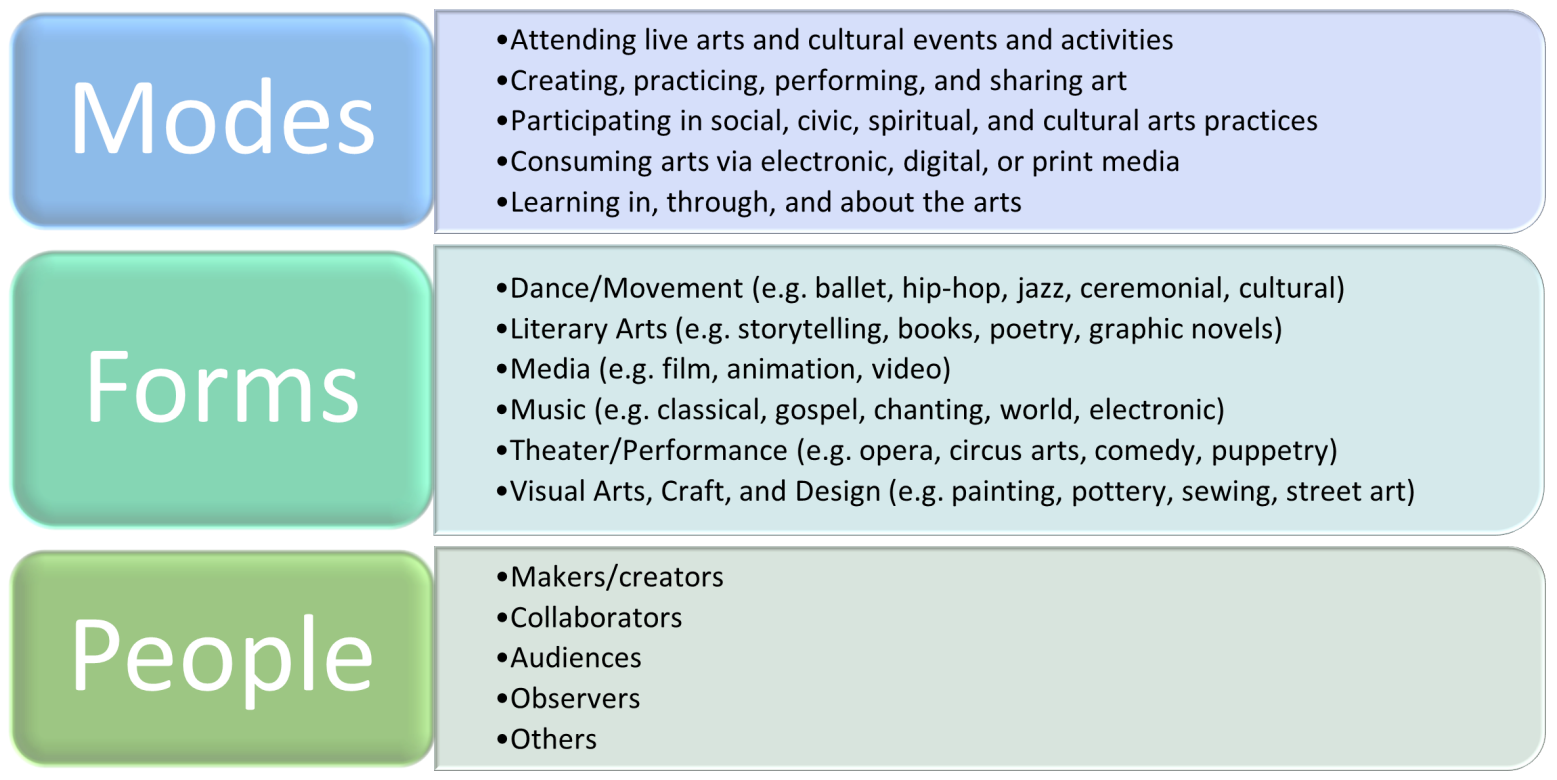
Defining “arts and cultural engagement” according to the definition by Sonke *et al.* (2023).

ACEng is a complex human behaviour involving dimensions related to modes of engagement (ways in which people engage, including informal, formal, live, virtual, individual, and group participation), forms (art forms or disciplines with which people engage) and people (makers/creators, collaborators, audiences, observers, and others) ( Sonke *et al.*, 2023) ( Figure 1). It encompasses formal and informal activity within many different domains of human life, including artistic practices, cultural traditions, and everyday creativity.

Numerous studies have documented individual-level barriers to ACEng. For example, The Cultural Barometer Surveys conducted across multiple European countries routinely identify socio-demographic factors predicting engagement with cultural institutions (e.g. going to museums, concerts, libraries, cinemas or watching cultural programmes on TV). They focus on factors such as age, education, and socio-economic status as well as mentioning geographical proximity. Barriers are largely focused on individual factors (time, cost, interest, information, access) with a small focus on choice and quality ( European Commission, 2017; European Commission, 2013). Reports such as this typically focus on the fact that large numbers of respondents mention these factors as barriers to access (e.g. >80% of respondents in each EU member state mentioned at least one of the individual barriers in the Eurobarometer Survey) ( European Commission, 2017). These reports, alongside broader policy-making processes, have led to the promotion of better access, inclusion, and wider participation in arts and culture, whereby the primary responsibility for increasing such engagement has tended to be on arts and cultural institutions. For example, the European Expert network on Culture highlighted a range of so-called ‘downstream’ solutions to increase and diversify audiences, including altering communications strategies, improving customer relationship management, changing pricing, and running outreach programmes ( Bamford & Wimmer, 2012). However, over the past forty years, even though there has been substantial pressure on arts organisations to broaden their audiences, there has been little variation in attendance rates across many countries such as EU nations ( O’Hagan & Castiglione, 2010). This suggests that these current efforts of tackling so-called downstream factors affecting individuals’ engagement are not working. Further, when looking at national participation rates across countries, even after accounting for individual-level factors, there are still large national differences in rates of ACEng ( Falk & Katz-Gerro, 2016). In other words, other factors operating at neighbourhood or societal levels are also determinants. Consequently, focusing solely on so-called ‘downstream’ predictors is insufficient and unlikely to lead to any meaningful change in rates or equity of ACEng.

Research from a vast array of disciplines has provided more nuanced detail on what drives arts and cultural engagement from the perspectives of both production and consumption. For example, psychological research has shown the influence of education, childhood experiences, parental background and personality on interest and engagement in the arts ( Vartanian & Goldstein, 2020). Cultural anthropology has explored how differing ideas of aesthetic value in different societies shape patterns of cultural engagement across cultures and generations ( Sharman, 1997). Sociologists have shown that culture is embodied by individuals across the life-course as a form of ‘capital’ (actual and potential resources), with cultural engagement functioning as an expression of this capital ( Bourdieu, 1993). Behavioural science has explored how the expression of preferences through consumption behaviour by those who engage with arts and culture drives cultural production and consequently what cultural offering is available for people to engage with ( Colbert & d’Astous, 2021). Arts management research has shown the role of internal management and marketing of cultural organisations in affecting consumer behaviour ( Chong, 2009). Creative and cultural industries research has shown how the structure of the labour market for artists and the demographics of its artists guides what art is produced and therefore which demographics are represented in contemporary culture ( Brook *et al.*, 2020; Carey *et al.*, 2021). Urban geography research has identified how the location of cultural facilities and distribution of arts employment can affect spatial patterns of cultural engagement ( Evans, 2016). Ecological approaches have shown that culture is a dynamic organism involving complex interdependencies between demand and production, across commercial, publicly-funded and domestic spheres ( Holden, 2015). Cultural policy approaches have demonstrated how country-level factors like cultural policy models, national political ideology, welfare regimes, GDP per capita, unemployment levels, levels of inflation, and levels of happiness as well as chronological factors and events such as economic upheavals are all related to ACEng ( Rius-Ulldemolins *et al.*, 2019; Srakar *et al.*, 2018). Economics approaches have shown that ACEng at an individual level is part of a broader ecosystem incorporating training and employment levels within the cultural sector, cultural imports and exports, and funding from governments, private sector and individual households ( Srakar *et al.*, 2018). These are just some examples of a broad and diverse multi-disciplinary literature highlighting that, far from barriers being constructed at an individual so-called downstream level, predictors of engagement also involve larger so-called ‘upstream’ social, societal, economic and political factors.

Yet despite such a large body of theory and evidence, this work has largely occurred in disciplinary silos, which has led variously to contrasting discourses and approaches, criticism, and inconsistent findings. For example, an EU report on policies and good practices in public arts and cultural institutions focused on addressing physical, financial, geographical and intangible barriers as solutions for improving access ( OMC, 2012). But this work was criticised for failing to acknowledge the complexity of the whole system. It theorised ACEng within the context of a relationship between audiences and cultural institutions, but this understanding is not all encompassing. Many acts of individual creativity do not involve interactions with cultural institutions or publicly supported provision ( Miles & Gibson, 2016). Further, bottom-up, grassroots initiatives such as so-called ‘bedroom artists’ and ‘guerrilla art’, not to mention online coproduction of art, are emerging types of ACEng that are clear moves away from institution-led arts activity. They rely on a dual identity between individuals as creators and audiences as well as co-creative processes ( Tomka, 2013). So, addressing barriers by working with arts organisations in isolation from the broader context they operate in is not sufficient to tackle challenges in ACEng. There is also increasing recognition that the strength of some individual-level factors is moderated by so-called ‘upstream’ factors. For example, income and education may play less important roles as determinants of engagement in countries that have respectively more generous state arts subsidies or a relatively highly-educated labour force ( Coulangeon, 2005; Katz-Gerro, 2004). However, this only occurs in some countries (e.g. Scandinavian ones), highlighting that other variables are moderating this relationship ( Falk & Katz-Gerro, 2016).

What we lack is a rigorous comprehensive understanding of these determinants that goes beyond descriptively showing inequalities, instead explaining why these inequalities exist and how they can be overcome. To achieve this, this research set out to construct an interdisciplinary theoretical framework synthesising the existing knowledge on determinants of arts and cultural engagement that could be used to ground and guide future research studies and new directions in policy and practice.

Bringing together diverse theories from multiple different disciplines raises some important issues. For example, extracting theories from their original disciplinary framework can affect how they are understood and interpreted, risking the result of ‘context-free theories’ that do not make sense out of their original context. Further, there can be semantic issues - similar terms can mean very different things in different disciplines, which can lead to confusion. However, there are also opportunities for tackling complex multidisciplinary challenges: by comparing and integrating findings, accelerated progress can be made in understanding and addressing research challenges and in developing new theory that sits comfortably across multiple different disciplines. Therefore, to provide support to such a large-scale theoretical project, we identified three complementary theoretical bases to use as our guiding lens (outlined below) and employed a clear methodological approach, which included developing an ‘apparatus’ of terms and concepts that guided the structure of the framework. Further, we drew on the expertise of a multi-disciplinary group of researchers, who fed into the development of the framework and critiqued it from their respective disciplinary perspectives.

### Theoretical bases

While there are many variations of social-ecological theories, two were particularly foundational to the work presented here.

Ecological systems theory - developed initially by psychologist Urie Bronfenbrenner and elaborated by sociologists and ecologists since – provides a basis for conceptualising the dynamic interrelations between individuals, communities, and the environment ( Bronfenbrenner, 1977; Bronfenbrenner, 2000; Kilanowski, 2017). Social-ecological models that have emerged from this theory represent a convergence of input from psychological, biological, social, mathematical and physical sciences and situates individuals and their behaviours as firmly rooted in their environment. These models differentiate between the microsystem (factors closest to the individual affecting their behaviours), mesosystem (neighbourhood interactions), exosystem (community contexts and wider social networks), macrosystem (societal and cultural values and influences), and chronosystem (which incorporates time, historical context and policy). This framework has previously been used to understand behavioural determinants and outcomes not just within its original domain of developmental psychology but also within a wide array of health and social interventions ( Bronfenbrenner, 2000; Kilanowski, 2017; Sallis *et al.*, 2015).

Relatedly, ecosocial theory – developed by epidemiologist Nancy Krieger – focuses on what drives changing patterns of social inequalities, challenging an over-focus on individual level determinants. Ecosocial theory has a particular focus on health, but arguably has a relevance to ACEng as arts behaviours are increasingly being acknowledged as health behaviours ( Rodriguez *et al.*, 2024). Ecosocial theory builds on ecological systems theory in seeing people and their behaviours as part of populations - dynamically shaped by and shaping their environment - and in considering the influence of geographical, historical and spatiotemporal factors. But it argues in particular for the influence of macro-level phenomena as driving and constraining meso- and microlevel phenomena. Ecosocial theory proposes that people literally embody, biologically, their lived experience in societal and ecological context. Consequently, attempting to change individual behaviours without addressing the societal context is fundamentally flawed.

Additionally, social-ecological theories have been deeply influenced by work from complex adaptive systems science (CASS) ( Preiser *et al.*, 2018). CASS focuses on understanding the principles of interactive and dynamic systems that change over time. In previous work, we have argued that arts engagement is a ‘complex’ activity in that it involves multiple interacting components that operate at individual and group levels ( Fancourt *et al.*, 2021). As such, following the principles of complexity science, there will be multiple simultaneous interacting factors that enable or hinder behaviours, involving positive and negative feedback loops, bidirectionality and non-linearity in effects, disproportionate relationships (whereby small changes to one factor can have large effects elsewhere within a system), and emergent outcomes (whereby new barriers or enablers can emerge through the interaction of different factors within a system) ( Byrne & Callaghan, 2013; Preiser *et al.*, 2018; Rogers, 2008).

In view of this landscape, this study aimed to leverage the large volume of research and theory on determinants of arts and cultural engagement from diverse disciplinary domains and use the theoretical foundations of ecological systems theory, ecosocial theory, and complex adaptive systems science to develop a new theoretical framework: the fRAmework of the DetermInants of Arts aNd Cultural Engagement (RADIANCE). RADIANCE aims to ‘shine a light’ on the determinants of ACEng. Such a framework is essential to support individuals, groups, and organisations operating within the ecosystem of ACEng to identify and breakdown barriers, thereby improving equity of access and the opportunities for equity of individual and societal benefits of engagement.

## Methods

**Figure 2.**
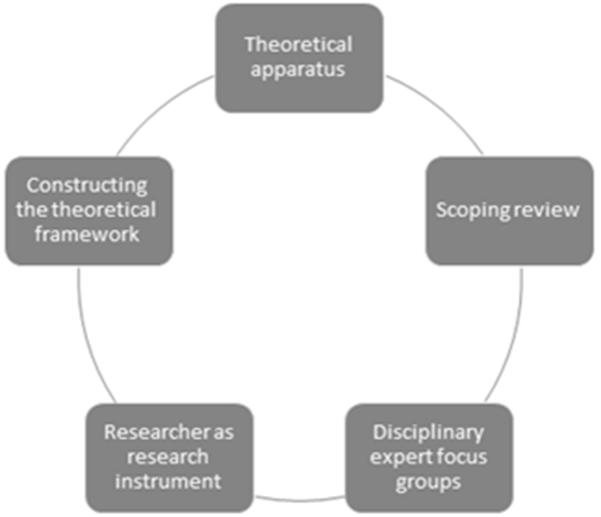
Visual representation of our 5-step iterative process, whereby we engaged in systematic processes of theorising in order to construct the RADIANCE framework.

In line with Swedberg (2012), we engaged in processes of theorising in order to construct a new theoretical framework that could be operationalised and explored within future empirical research. We approached the processes of theorising as systematic within a ‘heuristic of social science’: an approach to discovering new ideas ( Abbott, 2004). We engaged in an iterative 5-step process to engage in theorising that led to the construction our RADIANCE framework (See Figure 2). Across the steps, we worked as a team to co-construct factors that are conceptualised as determinants of ACEng. We moved back and forth between different steps until we reached consensus among our team that our framework met the aims of our research.

### Step 1: Constructing a theoretical apparatus

First, we applied the ‘search heuristic’, which involves borrowing a “whole apparatus” from one discipline and applying it to another to create new knowledge and innovate in a way that has never been done before ( Abbott, 2004). In our case, we drew upon multiple disciplines to do this, constructing our “apparatus” as ways of thinking from a range of interdisciplinary theoretical histories, considering how these theories and models may be relevant to innovate in the study of ACEng. This apparatus was more specific than the theoretical bases identified above and focused on identifying some key terms and concepts that could be sensitively applied within the construction of our framework to act as a skeleton for future steps in the process (see Table 1).

**Table 1.** Our theoretical apparatus.

Name of theory/model	Description	Key terms and concepts
**Social-ecological model** ( Bronfenbrenner, 1979; Crawford, 2020)	Originally conceived within the context of child development. Theorises the child as situated within a broader ecosystem of multiple levels, exploring how these different social levels affect development. We selected this model as a strong foundation and underlying structure for the theoretical framework as a whole.	• *Microsystem:* A person’s immediate environment, including day-to-day interactions and interpersonal relations • *Mesosystem:* “A system of microsystems” e.g., how school and home interact to create a mesosystem • *Exosystem:* Microsystems interacting in ways that affect the individual, but do not contain them e.g., the neighbourhood • *Macrosystem*: The culture and social structure • *Chronosystem:* Developments that occur across the lifetime
**Behaviour Change Wheel** ( Michie *et al.*, 2011; Michie *et al.*, 2014)	Behavioural science provides insight into how to identify facilitators and barriers to particular behaviours. Specifically, the behaviour change wheel is a ‘behaviour system’ known as COM-B, involving three conditions (capability, opportunity, and motivation) that can be used to create interventions to work towards behaviour change. Primarily used in the context of changing health behaviours. We selected COM-B as a clear structure to organise and categorise many of the diverse micro-level factors that have been identified as influencing an individual’s ACEng.	• *Capability:* Physical (e.g., skills) and psychological (e.g., knowledge) capabilities to perform a behaviour • *Opportunity:* Physical (e.g., afforded by the environment) and social opportunities (e.g., afforded by interpersonal influences) for a behaviour to occur • *Motivation:* Reflective (e.g., intentions and evaluations) and automatic (e.g., wants and needs) motivation to perform the behaviour
**Field theory** ( Bourdieu, 1993; Pierre Bourdieu, 2005)	Fields are theorised as the context or framework of a particular discipline, practice or subject, whereby one’s position within the field is determined by habitus (the skills and resources that govern how people engage with the world) and capital (actual and potential resources). We used field theory to understand how individuals are positioned within their wider contexts and the tangible and intangible benefits they can accrue from ACEng.	• *Social capital:* Resources from social networks and relations • *Cultural capital:* Having knowledge of culture, a kind of ‘cultural competence’ ( Johnson, 1993) • *Symbolic capital:* Acquisition of prestige and reputation • *Economic capital:* Assets considered in monetary terms
**Social integration** ( Berkman *et al.*, 2000; Durkheim, 1995; Durkheim, 1982; Durkheim, 1997)	Analysis of the processes by which societies can maintain social integration and solidarity. Includes a theorisation of a ‘collective consciousness’ that encompasses shared norms and values that holds groups together. We anticipated a large number of social factors that could act as determinants of ACEng, so selected this model as a way of differentiating some of the key social determinants.	• *Norms:* Social facts that shape thoughts and behaviours • *Values:* Relating to morality, socially constructed principles • *Social networks:* the web of social relationships that surround an individual and the characteristics of those ties • *Social cohesion:* Strong bonds and the absence of social conflict • *Social change:* the process by which society and social interactions change over time

### Step 2: Literature review

We then conducted a scoping review to explore what empirical literature exists that may have already identified factors that could be theorised as determinants of ACEng. This included searching for literature that was: 1) using the language of determinants within the context of ACEng; 2) using different concepts or language in the context of ACEng that could be theorised as determinants; and 3) using the language of determinants in the context of other kinds of social engagement that could be relevant to ACEng. This was a very broad scoping exercise, and we drew on inspiration from Arksey & O’Malley (2005) in our approach, searching relevant databases ( Google Scholar, UCL Explore, PubMed, ScienceDirect, JSTOR, Web of Science) for English-language papers published in peer-reviewed journals since 1950, and charting the relevant studies that we found into an excel spreadsheet v2403 **(see search terms in Extended Data [ Fancourt & Warran, 2024]).**

### Step 3: Disciplinary expert meetings

**Figure 3.**
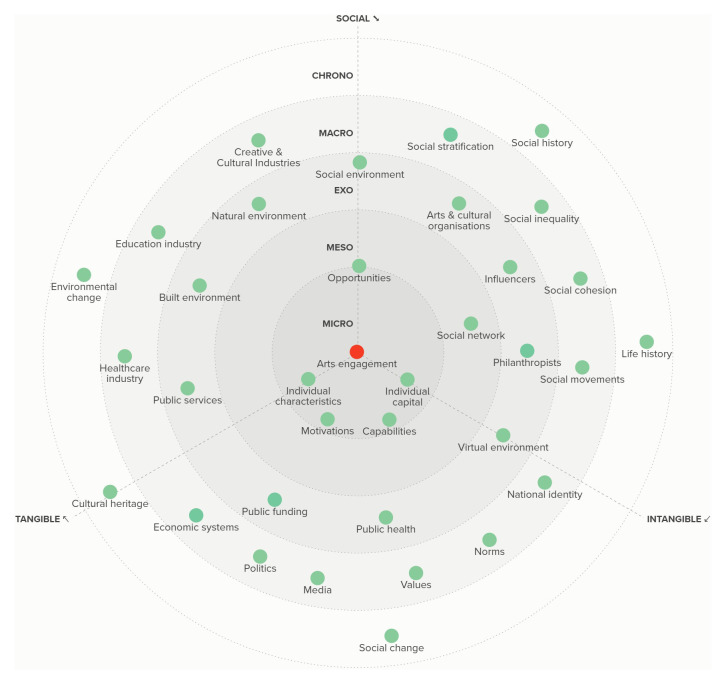
The RADIANCE framework of determinants of arts and cultural engagement.

Complementing our scoping review, we convened a “disciplinary expert steering group” consisting of 11 academics with expertise within and across sociology, social psychology, clinical psychology, education, cultural history, the arts, behavioural sciences, health humanities, art history, philosophy, geography, and politics who we identified as being able to meaningfully explore this work with us. We held a series of group meetings, discussing relevant theories and factors that may relate to determinants of ACEng, in addition to asking about barriers and enablers to engagement (*** see meeting agenda in Extended Data***). These meetings took place concurrently with our literature reviewing and exploration of pre-existing theories, whereby we looked across the key factors emerging and discussed them within the meetings. With permission from the academics in the meetings, we recorded the discussions and used the auto-transcribe feature of Microsoft Teams, alongside making extensive notes during the meetings. We used the transcripts and our notes to identify key literature from the discussions that could be used to inform the development of our framework and added notes to the spreadsheet formed in Step 2. In addition, after completion of our framework, we sent the draft of this manuscript with Figure 3 to those who had participated in the meetings, integrating additional feedback from these academics that was submitted to us via email.

### Step 4: Researcher as research instrument

We drew upon qualitative understandings of ourselves as research instruments ( Merriam & Tisdell, 2015), whereby we engaged in our own reflexivity and team discussions to engage together in collective theorising in view of our research aims. We engaged with our notes from the disciplinary expert meetings, literature sourced through our scoping review, and theoretical apparatus, “filtering out” elements that were “not central” to our focus, and “filtering in” those that were relevant ( Schwartz-Shea & Yanow, 2012). We had monthly group discussions across a 10-month period throughout the development of the work that included DF (professor with a background in psychobiology and epidemiology), KW (research fellow with a background in qualitative social science), and a team research fellow (with a background in history and global health). 

### Step 5: Constructing the theoretical framework

Through our discussions, we engaged in digital diagramming using the software Kumu version 3.3 (an online mapping tool) to work together to visualise the factors that we had co-constructed as determinants of ACEng through steps 1-4. This involved iterative discussions between the team and disciplinary experts using different forms of visualisation until consensus was reached that the visualisation represented our processes. We refined the language of factors and combined those that were similar, engaging in the abductive process of reflecting on pre-existing theories and making changes based on emerging findings from our discussions. As a ‘product’ rather than a ‘process’, the visual representation became our ‘framework’. We chose the language of ‘framework’ as it could be used as a ‘lens’ in which to study phenomena ( Anfara & Mertz, 2006). A framework builds on the concept of ‘theory’, understood as a set of interrelated constructs that explore relations among factors that can be used to explain or predict phenomena ( Anfara & Mertz, 2006). This met our aim of creating a tool to guide future research, policy and practice exploring the determinants of ACEng.

## Results

Overall, we identified 35 different factors that can act as determinants of ACEng across micro, meso, exo, macro and chrono levels. We broadly categorised these as social (i.e. a primary feature being the interaction of people), tangible (i.e. a primary feature involving physical assets or resources or the production of physical assets), and intangible (i.e. constructs that do not have a primary physical basis but instead have a virtual or imaginary basis). The determinants are depicted in Figure 3.

An overview of each factor is provided below. **A full description of each factor and summary of how it interacts with other factors in the figure is provided in the Extended Data ( Fancourt & Warran, 2024).** Box 1– Box 3 provide examples of how RADIANCE can be applied to better conceptualise determinants of ACEng.

**Box 1. Case study 1**

**Audience development initiatives in the arts**

Since the late 1980s, there has been a growing focus in cultural policy and arts organisational practice on audience development (aka audience participation and audience engagement) – deepening relations with existing audiences and building new audiences, seeking to expand the reach and depth of arts engagement ( Arts Council England, 2017). Yet, despite increased audience development initiatives across this spectrum in the arts and cultural sector over the last several decades, longitudinal data and analysis suggests only a limited degree of change in diversity of engagement ( Hadley, 2021). Many audience development initiatives have focused primarily on factors that sit within the micro to exo levels of our RADIANCE framework such as individual behaviours ( capabilities, opportunities and motivations) and needs or the practices of arts and cultural organisations such as marketing, ticketing practices, commissioning and programming ( Arts Council England, 2017).

**Figure 4.**
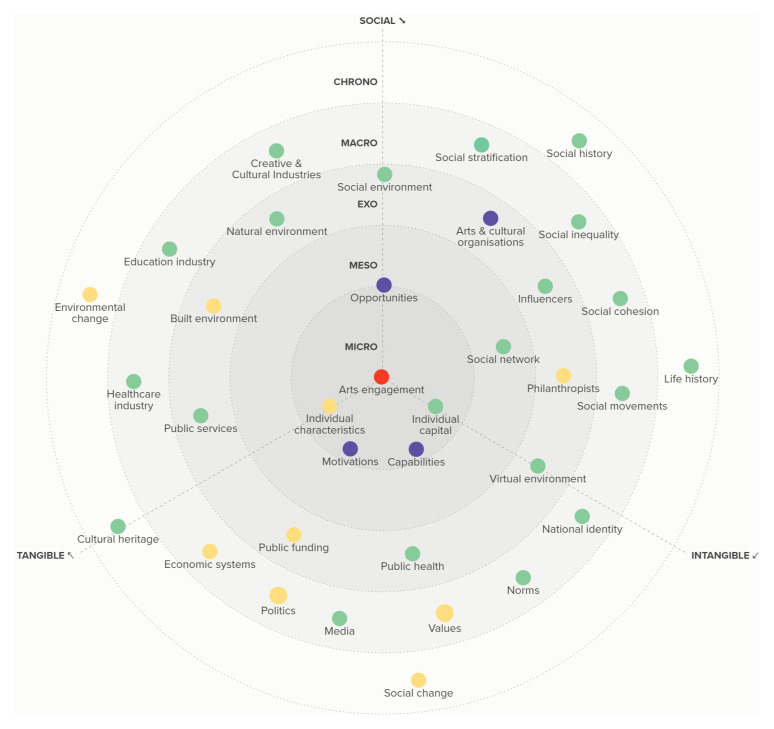
Example factors considered in some audience development initiatives as described above (shown in purple), and some of the wider factors that needed to be taken into account but were not (shown in yellow) to increase arts engagement (shown in red).

This approach has previously been criticised as reductive ( Hadley, 2021; Warran, 2021). RADIANCE highlights how not taking into account further factors are likely responsible for the limited effects of these initiatives (see Figure 4). For example, schemes that focused on reducing admission fees to arts experiences failed to take into account how micro-level financial circumstances ( individual characteristics) are also deeply interconnected to macro-level economic systems such as employment patterns and social change such as economic uncertainty and politics. These could have not only directly affected ability to purchase tickets but also influenced mental health and leisure time available, simultaneously reducing individual capabilities, opportunities and motivations. Further, economic systems affect the money that governments have available to publicly fund the arts ( public funding), thereby meaning that arts organisations have to operate on commercial models or rely on philanthropists, potentially changing priorities away from social justice models of audience development and focusing on increasing ticket sales that may mean programming ‘less risky’ artworks that reach out to ‘safe’ audiences, potentially creating barriers to diversifying audiences. Changes to ticketing also fail to take into account other financial issues relating to attending such as public transport ( built environment), especially given environmental changes such as climate change that could affect values around alternative transport such as driving.

**Box 2. Case study 2**

**The COVID-19 pandemic and arts and cultural engagement**

**Figure 5.**
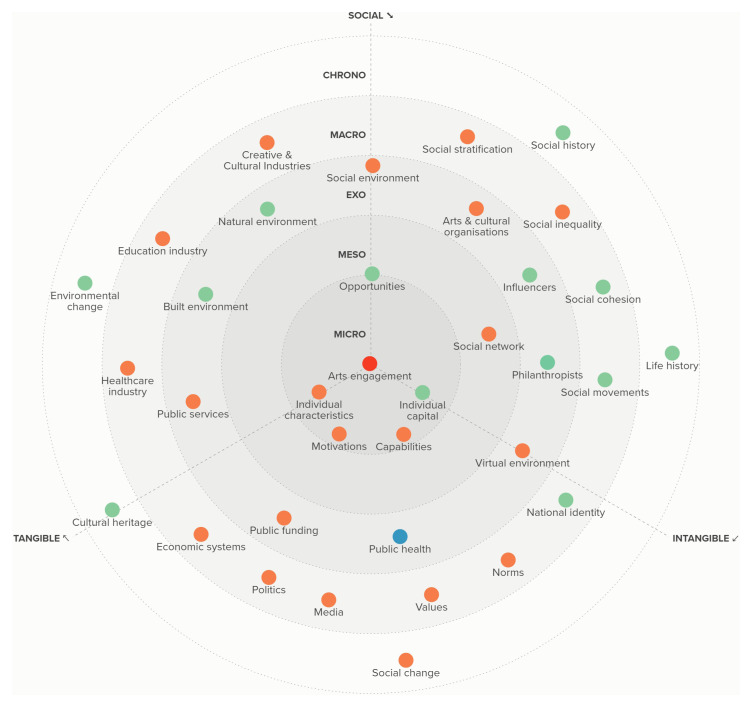
The widespread effects of the COVID-19 pandemic (a public health challenge; shown in blue) on factors across the ecosystem (shown in orange) that led to changes in arts engagement (red).

The COVID-19 pandemic was, at its heart, a public health issue that resulted in major shifts in people’s arts and cultural engagement patterns ( Arts Professional, 2023) (see Figure 5). However, RADIANCE highlights how the effects of it reverberated across the ecosystem, leading to impacts across so many factors that there was no simple solution to enhance engagement once more. For example, the social change caused by COVID-19, including national lockdowns, radically affected people’s social behaviours, including their engagement with their social networks, the make-up of their social environment, and their opportunities to engage. While there was increasing creative engagement at home during lockdowns ( Bu *et al.*, 2022; Mak *et al.*, 2021), and developments in virtual environments for arts engagement, there were also problems such as venue closures and job losses in arts and cultural organisations ( Owen *et al.*, 2020), and detrimental effects on the education industry including school closures, which reduced children’s arts engagement. The pandemic also affected societal and individual values, including how people wanted to spend their time. Some people only wanted to attend events that ‘guarantee a good time’ and not take as many ‘risks’ as pre-pandemic times due to economic uncertainty, leading to changes in motivations to engage and behavioural norms ( Reece, 2023). Data from The Audience Agency shows that even post-pandemic, more people are tending to book for arts events at the last minute than before the pandemic ( Arts Professional, 2023).

The pandemic also impacted on economic systems, which had implications for public funding for the creative and cultural industries and for the financial position of freelancers ( Reece, 2023). Indeed, research exploring the impact of the pandemic on freelance cultural workers showed shifts from ‘careerist’ values, relating to achieving and maintaining a career in the arts, to a greater emphasis on personal relations, such as family life, affecting the motivations of professionals, which in turn reduced the opportunities available for audiences to engage with ( Warran *et al.*, 2022). For people affected by COVID-19 (especially long Covid), capabilities either to produce or consume arts were additionally affected. Economic effects also influenced individuals. The pandemic and cost-of-living crisis that has followed have impacted individual finances ( individual characteristics, which in turn influenced financial capabilities), as well as increasing social stratification and social inequalities in relation to both workforce inequalities in the creative and cultural industries and who engages in the arts. All of this has led to a focus on impacts at the individual level of the ecosystem too, further affecting motivations to engage (e.g., consumer confidence and unpredictable booking patterns) ( Reece, 2023).

As such, a single change in a seemingly specific factor (i.e. public health) has resulted in widespread effects, leading to challenging changes in patterns of individual arts engagement. RADIANCE enables us to visualise these effects.

**Box 3. Case study 3**

**England’s Sing Up Programme (2007-11)**

Sing Up 2007-2011 was a UK government-supported Music Manifesto National Singing Programme that aimed to raise the status of singing and increase opportunities for school children throughout the country to enjoy singing as part of their everyday lives ( Sing Up, 2011). The programme was very successful: by 2011, Sing Up had engaged with over 95% of state primary schools in England.

**Figure 6.**
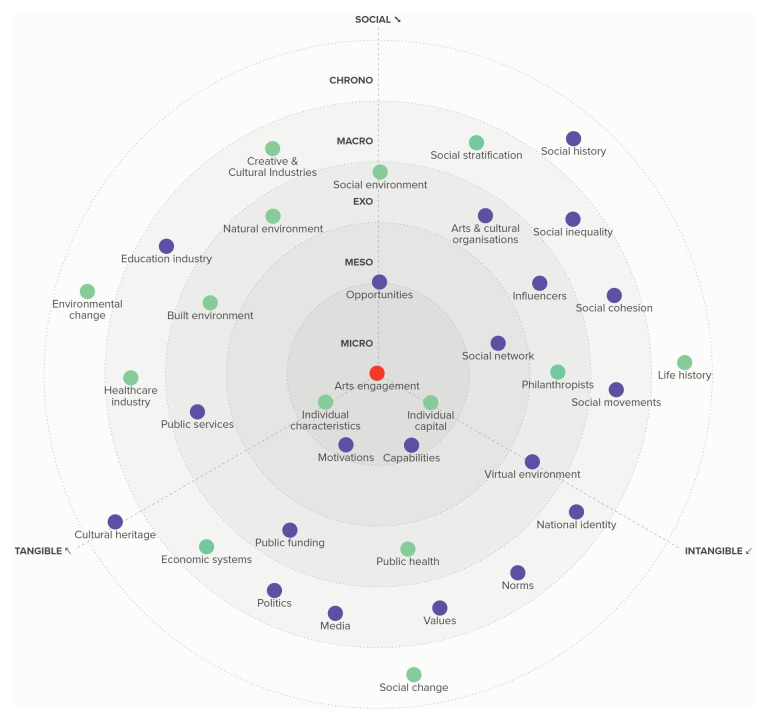
The diverse determinants considered and addressed (shown in purple) in England’s successful Sing Up programme.

The programme focused on enhancing engagement in schools (a public service; see Figure 6), but it took a wide lens, focusing on transforming singing in the entire education industry. The programme directly addressed children’s capabilities, opportunities, and motivations to sing through embedding learning, play and confidence-building. It made use of key resources, including a major online bank of resources ( virtual environment), including existing and new compositions that drew on England’s cultural heritage and social history. It also drew on influencers to promote the programme (including a national singing ambassador), partnerships with arts organisations, and widespread media campaigns. The programme also didn’t just focus on children, but on their wider social network, such as teachers and other students, building their capabilities, opportunities, and motivations to sing. Over 60,000 people in total were involved in Sing Up’s training and development activities. This created strong social cohesion amongst teachers across England, enhancing buy-in with the programme, and built a wider social network of singing leaders, creating a supportive social environment and leading to a national social movement of school-based singing. By taking a national approach and aiming for all primary schools to become ‘singing schools’, the programme sought to change norms and values around school singing and reduce social inequalities in engagement with singing. The buy-in of government ( politics) also enabled the ring-fencing of public funding and positioned singing as part of England’s national identity.

However, despite its success and the strongly networked way it was designed, it is notable that the programme was scaled down after 2011, after a change in government ( politics) led to the withdrawal of public funding. This demonstrates how although addressing multiple factors can be vital to enhancing ACEng, changes to individual factors can be sufficient to lead to substantial reductions in ACEng (see Case Study 2).

### Micro

At an individual level, engagement in the arts is directly influenced by our perceived capabilities to engage (our mental and physical capacities), as well as our motivations (automatic and reflective habits and beliefs), our individual characteristics (observable traits, personality traits and genetic factors) and our individual capital (economic, symbolic or cultural resources or assets).

### Meso

When considering the ‘system of micro-systems’ within which we operate, our ACEng is influenced by the opportunities we are presented with (whether social or physical), as well as our social networks (the number and characteristics of ties between us and other people and the size and density of the networks those ties create).

### Exo

As well as our own micro-system, we can also be influenced by other micro-systems. These include the location, access, and functional and aesthetic components of our built environment (the human-made structures, systems, and services that connect to place, such as buildings, spaces, centres), our natural environment ((the nature of the living space such as soil or water, as well chemical and physical properties, such as the climate and organisms) our social environment (the socio-demographic composition of areas, civic participation and engagement, crime and safety, discrimination, and trust) and our virtual environment (the infrastructure and opportunities for online interaction). Our public services also provide a network of organisations that support citizens in a range of ways, such as with health and education, as does public funding (money allocated for public goods and services that comes from the government). Arts & cultural organisations themselves (companies, businesses, associations, and groups that operate on commercial or non-profit models to provide artistic products, such as performances or exhibitions) additionally play a major role in our ACEng. Philanthropists (people who donate substantial economic capital, often alongside expertise or voluntary support, in support of a particular organisation or cause) and influencers (highly visible digital content creators who have substantial following) additionally influence patterns of ACEng. Public health (the health outcomes of groups of individuals and distribution of disease) can additionally exert an influence on ACEng.

### Macro

Our culture and social structures are also important determinants of ACEng. Patterns of social stratification (a socially constructed system affected by broader political, economic and social structural factors that enables the ranking of individuals and groups within societies based on ascribed or achieved traits), social inequality (the unequal distribution of valued goods or opportunities), and social cohesion (the extent to which there is connectedness and solidarity among groups) all have a role to play, although this can be challenged by social movements (forms of collective behaviour or collective action that attempt to change some aspect of society). National identity (the value ascribed by groups or individuals to a particular nation), norms (‘rules’ within a society or culture that influence social action), values (the constructed principles or ideals that make up cultural life) and culture (the set of distinctive spiritual, material, intellectual and emotional features of society or a social group) also play a role in determining ACEng. These can be enacted, reinforced or challenged by media distribution (forms of communication, such as the internet, broadcasting, advertising, and print), politics (activities or policies associated with government or administration, often relating to actions concerned with power, status or authority) and economy (processes or systems that produce, sell, and buy goods and services in a country or region). Industries including the creative and cultural industries, education industry and healthcare industry within countries further have influence on ACEng.

### Chrono

Developments across lifetimes can further affect ACEng (and the other level determinants). Social histories (the past explored through lived experiences), cultural heritage (viewing the history of human societies through arts and cultural activities and practices) and life histories (patterns of species growth, development, reproduction, and mortality) provide key contexts in which ACEng occurs. Social change (shifts in societal structures, practices or demographies) and environmental change (changes or disturbances to the environment through ecological processes) can additionally exert effects on the whole system of determinants.

## Discussion

The proposed RADIANCE framework posits that an individual’s ACEng is determined by large numbers of interconnected factors that operate at different levels of influence. The framework challenges previous approaches that have described or attempted to address barriers to ACEng at micro-levels. This move is in line with similar shifts that have occurred within other domains such as health, where attempting to address disease incidence via a limited focus on ‘proximate’ individual risk factors has been criticised for being methodologically individualistic (akin to biological reductionism) and led to marked moves towards more contextualised approaches ( Krieger, 1994). While there have been moves towards acknowledging and attempting to address broader societal determinants of ACEng in recent years, they have remained relatively siloed in approach. So, this framework provides an ecosocial lens that it is hoped will provide a broader perspective and context for future work on determinants of ACEng, in practice, policy and research. Before exploring these implications of the framework, we will explore how RADIANCE can be best understood in light of a range of guiding principles drawn from the theories that informed its development.

### Theoretical principles

First, drawing on ecological systems theory, the organising levels within the model are illustrated in the framework as micro, meso, exo, macro, and chrono. But drawing on ecosocial theory, these levels are considered metaphorical – organising principles – rather than ontologically real. Determinants within ‘lower’ levels are not necessarily nested within ‘higher’ levels ( Krieger, 2011). As has been demonstrated repeatedly in public health, it is proposed that macro-level determinants are more likely to drive and constrain meso- and micro-level determinants ( Krieger, 2011). The reverse can happen under particular circumstances, as has been demonstrated in the work of individuals or small campaign groups influencing macro-level factors such as public funding. But we need to acknowledge and be cognisant of the challenge involved in effecting large-scale change from grassroots endeavours. This places emphasis on macro-level actors such as policy makers to engage actively in attempts to achieve the democratisation of culture.

Second, although this framework is organised in concentric circles, this is not intended to lend weight to ‘upstream/downstream’ or ‘proximal/distal’ metaphors. Indeed, we align our framework with ecosocial theory in seeing these metaphors as too temporally and spatially constrained – too discrete and sequential ( Krieger, 2011). Processes that affect ACEng occur simultaneously at different spatiotemporal levels (e.g. exo-level decisions on the formation of local grassroots arts community groups occurring alongside macro-level decisions on cultural funding policies), not necessarily sequentially. And so-called ‘distal’ factors, such as whether local libraries are shut down, have profound and immediate day-to-day effects on individuals, rendering the influence of them proximal (even if that influence is processed through exo and micro levels), as that processing happens so temporally fast.

Third, in relation to temporality, it is important to acknowledge that the timescale of political and economic institutional actions is considerably shorter than that of most ecosystems processes ( Hobbs, 1998). This has profound consequences in relation to the principle of ‘embodiment’ from ecosocial theory. We physically embody our lived experience in our societal and ecological context. So even if we address macro-level factors such as dramatically increasing funding for arts provision within local communities, this would not address the inequalities in arts engagement embodied and reproduced by adults who may have already grown up with limited ACEng opportunities across their childhoods, nor cultural values or norms that might influence individual motivations to actually engage. Even individual creative practices that occur aside from formal arts engagement are still influenced by formal institutions as they play a role in shaping macro-level factors like cultural values and norms that still influence individual practices. This helps to explain why macro-level barriers can be powerful for all kinds of arts and cultural practices, and enduring even in the face of major policy changes, and why lag times for real effects of macro-level interventions need to be properly accounted for.

Fourth, the framework is presented with an acknowledgement that not every piece of work on determinants of ACEng will be able to, nor need to, take account of every single factor presented. More localised work on specific clusters of factors is still crucial. Indeed, within social-ecological theory, ‘ecological niches’ are when certain factors converge together to form strong predictors to ACEng. Within the arts, certain niches have already been demonstrated, such as childhood, with childhood engagement acting as a significant predictor not only of whether a child then continues to engage as an adult but also whether they subsequently encourage arts engagement amongst their own future children ( Mak & Fancourt, 2021). The choice of particular niches may be driven by plans for particular interventions (e.g. arts programmes within schools or social prescribing schemes) or by theoretical interest. But it is hoped that this work can be contextualised within the broader framework that RADIANCE presents, and it is encouraged that RADIANCE is used to identify determinants that are related to the niche of interest and thus important to understand and explore alongside. In line with this, a number of existing smaller-scale theoretical frameworks have been incorporated into RADIANCE, as detailed within the methods section (such as the COM-B framework, social integration model and field theory). Others could also be mapped onto the framework. For example, Howard Becker’s concept of ‘art worlds’ recognises art as a collective activity and endeavour involving entire networks of people responsible for different stages in the process ( Becker, 2008), operationalised within RADIANCE as interactions between individual characteristics, capabilities, opportunities, motivations, social networks, influencers, arts and cultural organisations, philanthropists, and creative and cultural industries.

Fifth, RADIANCE provides an overarching framework for determinants of ACEng. But each individual’s experience will be unique: even if two people face very similar barriers to ACEng as a result of shared determinants at multiple levels (e.g. twins living within the same household), they may still have different actual experiences of these barriers due to differences both in specific micro-level barriers (e.g. different individual personality traits) and in how these individuals *adapt* to their environments. This principle of adaptation is crucial within ecological systems theory and explains, too, how individuals could change their arts behaviours even in the absence of any concrete changes in broader environmental determinants of their engagement through learning to navigate their environments more effectively ( Germain, 1978). Previous work applying the lens of ecosystems to understanding cultural engagement has documented how interconnections and interdependencies between individuals and organisations at different levels can develop organically to capitalise on available opportunities ( Gross & Wilson, 2020). In reverse, adaptation can be thwarted by chaos – the changing of multiple determinants in a way that disrupts and inhibits individuals from learning and adapting. Examples of chaos within determinants of ACEng include rapid successive changes in government policies before arts organisations have had a chance to adapt, such as in the case of Brexit in the UK, which led to confusion, complexity and detrimental effects on artists working in the creative and cultural industries ( Goodall, 2020).

Finally, RADIANCE is best understood through the principles of complex adaptive systems science (CASS). RADIANCE visually illustrates different determinants of ACEng, but it is the *interactions* and *relations* between these determinants that is most important: *how* changes in determinants such as the availability of arts and cultural spaces (assets) in the community influences individual-level ACEng ( Preiser *et al.*, 2018). These relations form dynamic networks of causal effects, which may not be linear or directly proportionate. Therefore, changes to small determinants could drastically alter ACEng, while large-scale changes (e.g. entire new cultural policies) may have very little impact at an individual level depending on the flow of interactions between different elements in the framework once these changes are made. To a certain extent, it is therefore very hard to predict and control the impact that changes to determinants will have. This emphasises the need for careful evaluation of interventions that attempt to remove barriers and the inclusion of check points within any interventions when changes could be made if planned benefits are not being found. Further, CASS challenges the very notion of individual determinants given all determinants are so inherently interconnected. In particular, the language of “barriers” is arguably inherently flawed given it implies that identifying and removing a specific obstacle will result in improvements to engagement, whereas in practice any attempts to focus on individual barriers in isolation from the broader ecosystem are unlikely to effect substantial change. Finally, although this framework brings together a large number of determinants, it is not, and could never be, complete, as this is not in the nature of complex adaptive systems ( Preiser *et al.*, 2018). There may be further determinants not captured in this model that could be incorporated into future versions, but there may also be new determinants that will emerge organically over time as the context of ACEng evolves. Consequently, theoretical work on determinants should remain an ongoing process.

### Implications for enhancing ACEng

RADIANCE has a number of implications for practice. First, contrary to previous reports and initiatives detailed in the introduction that focused on variations in ACEng due to individual characteristics (such as age, gender, ethnicity, socioeconomic position etc), RADIANCE posits that it is not these demographics themselves per se that influence engagement. Rather, these individual characteristics influence individuals’ capabilities, opportunities and motivations, either directly, or indirectly as they are perceived through patterns of social stratification. This process builds into the construction of values and norms that then influence capabilities, opportunities and motivations. This builds on previous empirical work, such as studies showing that poor mental health (an individual characteristic) influences ACEng through reducing capabilities such as self-esteem, cognitive functioning, and physical health ( Fancourt *et al.*, 2020). This has enormous implications for how to address barriers, as it suggests that the focus should be on identifying and addressing capabilities, opportunities and motivations (or factors in the indirect pathway such as social stratification) rather than individual characteristics themselves. This shift is important as such factors are modifiable, even though many individual characteristics that influence those factors (such as ethnicity) are not.

To address the more modifiable risk factors, we can apply insights from behavioural change ( Michie & Johnston, 2012), which has demonstrated that capabilities, opportunities and motivations can often be addressed through targeted interventions. For example, the behaviour change wheel (a tool for identifying and designing behaviour change interventions) shows that psychological capabilities can be enhanced through educational initiatives (such as providing information about how to engage with local arts and cultural activities) and training (such as providing lessons or workshops to develop artistic skills), while automatic motivation can be enhanced through enablement (such as making activities affordable or free) or modelling (e.g. running public campaigns showing people engaging) ( Michie *et al.*, 2011). As is clear, each of these proposed interventions draws in other meso-to-chrono-level factors: educational initiatives, training and enablement could involve arts and cultural organisations or public services such as schools, while modelling could involve media or influencers ( Michie *et al.*, 2011). All of these could be further supported by policy (such as publication and dissemination of new guidelines for education in schools or fiscal changes such as tax breaks for arts organisations providing enablement activities) ( Michie *et al.*, 2011). Consequently, even in the absence of necessarily knowing what the meso-to-chrono-level drivers of individuals’ capabilities, opportunities and motivations for ACEng are, applying the behaviour change wheel means we can deduce which meso-to-chrono-level factors within RADIANCE are theoretically expected to change an individuals’ capabilities, opportunities and motivations. These identified meso-to-chrono-level factors can then form the targets for research or evaluation. However, while it is accepted that many of the effects of meso-to-chrono-level barriers may be mediated via their influence on capabilities, opportunities and motivations, it is not to be assumed that this is always the case. RADIANCE deliberately does not include causal arrows between different factors as insufficient work has currently been done to demonstrate which factors should be linked and which direction these arrows should go. Further, it may be that interventions that affect ACEng are initiated deliberately or inadvertently at broader levels (e.g. unexpected chrono-level events or macro-level policy changes).

A further clear takeaway for practice is that attempting to change ACEng is complex. Behaviour change science differentiates behaviours that involve simple specific actions from those that involve more complex sequences that may need sustaining over time, which we argue ACEng is ( Michie & Johnston, 2012). Not only will intervening at one individual factor in the absence of intervening simultaneously on other factors be unlikely to exert change (especially meaningful or long-lasting change), but even if multiple factors are intervened upon together, they may be insufficient if necessary related factors are not addressed. For example, even at a micro-level, it is widely acknowledged that intervening strongly on knowledge or attitudes (capabilities) is insufficient if motivational processes are not simultaneously addressed ( Sniehotta, 2009; Webb & Sheeran, 2006). At larger levels, it is increasingly being argued that without transformational and structural changes, increased surface-level representation of individual groups such as minoritized ethnic groups or women or people with disabilities is meaningless ( Hadley *et al.*, 2022; Hill & Sobande, 2018). Relatedly, it is also important to remember that ACEng is not only a service that is provided for individuals. There has been an increase in the democratisation of ACEng in recent years, with individuals and grassroots organisations developing new forms of artistic expression and providing engagement opportunities relatively autonomously of many of the traditional ‘gatekeepers’ ( Tomka, 2013). Consequently, we recommend that work to enhance ACEng does not fall exclusively to arts and cultural organisations but that it becomes a priority for individuals and organisations working across all factors included in RADIANCE, with careful and diligent consideration of where barriers may be occurring (and how they intersect with other barriers) at all levels. This approach aligns with the social ecological model, which posits that in order to achieve and sustain behavioural change, action needs to be taken at multiple levels of the model at the same time, avoiding a bias towards focusing on micro-level factors ( Bronfenbrenner, 2000). It is also likely to become more feasible as work identifying the relevance and value of ACEng to diverse sectors continues to be undertaken ( Crossick & Kaszynska, 2016).

### Implications for monitoring ACEng

While policy is one of the factors that should be considered alongside others to reduce barriers, there are some additional considerations for policy in how we monitor ACEng. First, RADIANCE could be used to support endeavours to map national rates in ACEng. Some of this work is already being done in relation to the EU, where indicators on measurable aspects of meso- and macro-level factors relevant to certain forms of ACEng such as going to cultural venues (museums, cinema, cultural facilities etc) have been developed. These indicators include the built environment (e.g. transport), creative and cultural industries (e.g. creative economy), public services (e.g. arts education), social cohesion (e.g. tolerance and trust), and policy (e.g. governance quality) ( Montalto *et al.*, 2019). This work has already led to some mapping of how ACEng varies according to these factors and specific calls to identify how further macro-factors such as policy influence these outcomes ( Veal, 2023). It also has a relevance for the achievement of article 27 (the human right to enjoy the arts, United Nations General Assembly, 1949), because if ACEng can be measured internationally in relation to factors that could be barriers or enablers, more in-depth analyses will be able to be undertaken to ascertain what level of influence these factors each have. There are valid concerns about the challenges in undertaking work mapping engagement across countries and cultures, particularly given differences in definitions of ACEng and cultural practices ( UNESCO, 2012). However, RADIANCE could be used as a theoretical basis for any such work, to propose culturally-relevant determinants of ACEng that could be measured and to provide a framework against which findings can be mapped and conclusions about equity of access drawn.

Naturally, how the specific barriers identified in this framework are tackled will depend on the cultural policy and socio-economic conditions of respective countries. Countries vary enormously in the approaches they take to cultural policy, from weak to strong state intervention and whether individuals are seen as passive recipients of arts and culture vs active participants or generators ( Rius-Ulldemolins *et al.*, 2019). Specific cultural policy ideologies – i.e. what role policies advocate for the arts such as promoting excellence, social walfare, or political goals - can also affect how barriers could be addressed such as how policy components (e.g. tax incentives vs subsidies) could be leveraged ( Falk & Katz-Gerro, 2016). Similarly, the socio-economic conditions of countries will constrain any potential policy initiatives that could be taken. So, any conclusions drawn about how different factors influence ACEng will have to avoid leading to narrow “solutions”. Additionally, an important step in this work will be to extend the current focus on the EU to a broader international context (including low-income and global south countries), where there could be greater variation in some of the factors within the RADIANCE framework.

### Implications for researching ACEng

RADIANCE also has clear implications for research. Within quantitative research, the lack of a comprehensive understanding of the factors affecting people’s ACEng has led to confusion about how to control adequately for confounding factors when undertaking observational analyses (i.e. the factors that could in fact explain any link identified between ACEng and various outcomes such as health, wellbeing, education, and behaviours). To date within epidemiology, much of the focus of confounding bias when exploring the relationship between ACEng and these outcomes has been on individual-level factors such as socio-economic position ( Fancourt & Steptoe, 2019). However, by highlighting the range of other factors at meso-to-chrono levels that influence ACEng, RADIANCE demonstrates that there may be other confounders to be considered in epidemiological analyses. This understanding of confounders can also be used to inform recruitment into research studies by highlighting factors that could predispose individuals to take part in ACEng (and thus sign up to a study on ACEng), thereby helping researchers to design recruitment strategies that allow diverse samples to be identified and enrolled. RADIANCE provides an importance advance on our consideration of potential moderating factors by highlighting a much broader range across multiple different levels that could be explored as future moderators to enhance our understanding not just of what impact ACEng has on various outcomes but *for whom*. This could also help to assess the ecological validity of findings on ACEng and the outcomes of ACEng in studies – whether the findings are relevant to populations beyond those being studied.

RADIANCE also has implications for qualitative research designs. Within qualitative research exploring ACEng, there tends to be a focus on individual, subjective experiences, aligning with the ethos of qualitative research as suitable for capturing the emic, as opposed to the etic, perspective ( Williamon *et al.*, 2021). Yet, as qualitative research often focuses on individual experiences and perceptions of social reality ( Williamon *et al.*, 2021), this has, again, meant an over-emphasis on the micro-level of RADIANCE. There are qualitative studies that have looked at structural inequalities and consumption patterns in the arts, such as drawing on the work of Bourdieu ( Barrett, 2015; Turner & Edmunds, 2002; Whiting, 2021), and in recent years, there has been an emerging shift towards relational understandings of arts and cultural experiences that move out from individual-level motivations ( Tan, 2020), and take into consideration art-making based on human relations and their social context ( Bourriaud, 2020). But the connection between individuals and relations, and the broader macro social context is still limited. Whilst the micro-level is important to centre lived experiences from an equity perspective, it’s also important to recognise the role of factors at other levels of the system, as RADIANCE elucidates. Individuals embody broader structural factors, and it is possible to explore the ‘constitution of subjectivity’ ( Scharff, 2017). Aligning with this, RADIANCE supports with articulating more specifically what meso to chrono factors may be embodied by individuals within their personal, subjective narratives, supporting qualitative researchers to understand in more depth how and why barriers may manifest at an individual level.

### Applying RADIANCE

As demonstrated, RADIANCE has a number of clear applications within research, policy and practice. To further illustrate its utility, Box 1– Box 3 present worked examples showing how RADIANCE informs understanding of interventions and events that have affected ACEng in different contexts. Box 1 considers audience development initiatives applied in the UK that focused on marketing, ticketing practices, commissioning and programming and highlights how not taking into account further factors are likely responsible for the limited effects of these initiatives. Box 2 considers how a single change in a seemingly specific factor (i.e. public health) as a result of the COVID-19 pandemic resulted in widespread effects, leading to challenging changes in patterns of individual arts engagement. Box 3 considers an intervention that did successfully enhance ACEng in a particular population due to its successful consideration of multiple different determinants: the UK’s national SingUp programme (2007-11).

### Limitations

A strength of our RADIANCE framework is that it presents a usable model for future research and practice, supporting with articulating, understanding, and explaining the determinants of ACEng. Yet, as a conceptual tool, the factors within the model do not represent discrete, objective elements that are static. They have been conceptualised at a particular sociocultural moment based on current knowledge and theory and, aligning with ecosocial theory and complexity science, each factor within the system has the potential to evolve and change as our contexts and understandings do. The model is not, and can never be, ‘complete’. It remains for future research to continue to add factors to the framework and reconceptualise existing factors based on new and emerging research and knowledge that may come to the fore in the future.

Second, ecosocial theory explains that the interactions and relations between determinants are important to understanding barriers and enablers, not just the conceptualisation of the determinants themselves. Our framework currently presents different factors that can be conceptualised as determinants and highlights that there is a myriad of possibilities regarding how each element may interconnect or influence another factor within the system. However, it is possible to hone-in on particular factors in more depth and explore how changes within one specific factor of the system influence other factors. As such, future research could explore specific combinations of factors and how they influence one another to construct barriers or enablers, and how targeted interventions could be implemented in order to increase certain kinds of engagement. This research could build on the case study examples we have presented.

Third, the basis of our framework is that there are adaptive benefits of ACEng and that engagement with the arts is a human right. We therefore theorise that understanding the determinants of ACEng can support with increasing engagement which is a morally ‘good’ outcome. However, we recognise that there are complexities and counter arguments within this assertion. Notably, Steven Pinker is renowned for arguing that music is ‘auditory cheesecake’ with no selective evolutionary benefit and, thus, there is arguably no need to encourage engagement ( Pinker, 1999). Further, sociological theories have elucidated processes of legitimisation when it comes to what is ‘art’ and what is not ‘art’, founded upon structural inequalities ( Zolberg, 1990). Thus, it is not just considering what the determinants of ACEng are that is important, but also the determinants of what *kinds* of art. It is important for future research to use our framework to understand how social stratification may legitimise certain kinds of art as valuable in society, and how encouraging engagement in certain kinds of legitimated artistic experiences could exacerbate inequalities. Within this, our framework also does not consider aesthetics. Researchers from disciplines such as the arts and humanities may wish to apply our framework to understand how different factors within the model may work together to construct aesthetic values, linking to literature on conceptions of ‘genius’ and ‘quality’ ( DeNora, 2023; Heinich, 1997).

Interlinked to this, our framework is interdisciplinary, and this is a strength in that it combines different perspectives to explore determinants from multiple different theoretical standpoints. However, like all research, we have committed to certain theoretical constructs in order to operationalise our model within the context of understanding barriers and enablers to ACEng, for example using the language of ‘determinants’ and ‘factors’. This language may align better with certain disciplines than others, and those specialising in other disciplines may wish to explore how to apply RADIANCE to other theoretical lenses to explore different agendas. Such endeavours have been embarked upon within the wider application of the social ecological model (e.g., integrating it with the ‘relational turn’ in human geography to conceptualise different care practices; Bowlby & McKie, 2019) and such varied disciplinary applications could also be engaged with to adapt and apply RADIANCE within different disciplinary settings.

## Conclusions

There is a clear need to conceptualise determinants of ACEng beyond the individual level. Of note, amongst others, policymakers and stakeholders from the creative and cultural industries seek to understand better how to increase ACEng, but previous interventions targeted at the individual level have had only limited success. RADIANCE conceptualises 35 determinants of ACEng within a dynamic system, offering a more contextualised approach. It deepens theoretical knowledge by integrating insights from ecological, behavioural and complex adaptive systems theories to the context of the arts, and practice-based knowledge through theorising different factors within a multi-level system in a way that can be used to understand barriers and create targeted interventions to increase engagement in the future. It substantially advances understanding of why people engage in the arts. It is hoped that it will be applied empirically in future research to improve equity of access to ACEng, upholding the rights-based approach set out by the UN and ensuring that all those who wish to engage in the arts can.

## Ethics and consent

Ethical approval and consent were not required as the study involved no human participants. An expert steering group provided advice on relevant literature and theories, but did not partake in providing any data themselves.

## Data Availability

Transcripts from expert meetings cannot be shared as the advisory group is small and identifiable. Redacted transcripts of the expert steering group meetings that generated suggested theories and papers to include in the review are available from the corresponding author upon request. OSF: RADIANCE. https://doi.org/10.17605/OSF.IO/UAMXZ This project contains the following extended data: RADIANCE - ED - full definitions.docx (Full definitions and summaries of determinants within RADIANCE) RADIENCE - ED - search terms and meeting agenda.docx (Search terms and meeting agenda) OSF: PRISMA-ScR checklist for “A fRAmework of the DetermInants of Arts aNd Cultural Engagement (RADIANCE): integrated insights from ecological, behavioural and complex adaptive systems theories”. https://doi.org/10.17605/OSF.IO/UAMXZ (Fancourt, 2024). Data are available under the terms of the Creative Commons Attribution 4.0 International license (CC-BY 4.0).
