# Supplementary material for: A fRAmework of the DetermInants of Arts aNd Cultural Engagement (RADIANCE): integrated insights from ecological, behavioural and complex adaptive systems theories
Source: Wellcome Open Res. 2024 Jul 5;9:356. [Version 1] doi: 10.12688/wellcomeopenres.21625.1 (PMC11377924; doi:10.12688/wellcomeopenres.21625.1)
Supplement: Peer review history

# Reviewer report: Reviewer response for version 1

**DOI:** 10.21956/wellcomeopenres.23897.r91186

**Published:** 2024-09-05

**Authors:** Claudia Reinoso Araújo de Carvalho

**Affiliations:** 1 Universidade Federal do Rio de Janeiro, Rio de Janeiro, State of Rio de Janeiro, Brazil

The study is very relevant and presents very important results for understanding the determining factors for people's involvement in the field of culture and arts. Based on the theory of Eco ecological systems, Eco social theory and the science of complex adaptive systems, the authors developed a theoretical and methodological framework for understanding cultural artistic engagement. All reasoning and inferences are based on interdisciplinary references. The methodology is sufficiently described, as is all the theoretical support. The results are presented and discussed with rigor and complexity in the text. The study was well conducted, well written and will contribute enormously to informing future research in the field of culture, arts and other interfacing disciplines.

 Although the study refers to the United Kingdom context, I suggest the authors include some discussion about its applicability in different contexts. For example, in other countries.

 The proposal has great potential to be applied empirically in future research.

Is the work clearly and accurately presented and does it cite the current literature?

Yes

If applicable, is the statistical analysis and its interpretation appropriate?

Not applicable

Are all the source data underlying the results available to ensure full reproducibility?

Yes

Is the study design appropriate and is the work technically sound?

Yes

Are the conclusions drawn adequately supported by the results?

Yes

Are sufficient details of methods and analysis provided to allow replication by others?

Yes

Reviewer Expertise:

Associate Professor of the Occupational Therapy Course at the Federal University of Rio de Janeiro. Researcher in the area of ​​Cultural Accessibility. PhD and Master in Public Health

I confirm that I have read this submission and believe that I have an appropriate level of expertise to confirm that it is of an acceptable scientific standard.

# Reviewer report: Reviewer response for version 1

**DOI:** 10.21956/wellcomeopenres.23897.r91191

**Published:** 2024-08-06

**Authors:** Melissa Forbes

**Affiliations:** 1 School of Creative Arts and Centre for Heritage and Culture, University of Southern Queensland, Toowoomba, Queensland, Australia

Thank you for the opportunity to review this manuscript, which presents the research informing the RADIANCE framework for the determinants of arts and cultural engagement at the individual, community, and societal levels. This framework—the result of a rigorous multidisciplinary and multi-theoretical approach—illuminates the barriers and enablers to arts and culture engagements (“ACEng”). This framework is an important and much-needed contribution, with significant benefits to end users ranging from government policymakers and researchers to coalface arts and culture workers.

**Introduction**

 Considering evolutionary research, the authors argue that barriers to ACEng are unlikely biological; rather, they are likely to be social. The authors cite research that shows that "downstream" interventions to increase ACEng targeting individual factors have failed to increase ACEng. The authors draw on a vast array of multi-disciplinary research demonstrating that "upstream" barriers to ACEng interact in complex ways with downstream factors. The current study aims to overcome the disciplinary siloing characteristic of much previous work in this area.

**Method**

 The method is comprehensively described. The open-access extended data set, comprised of full definitions for the determinants in the RADIANCE framework, is a valuable resource.

**Results**

 Applying the framework to case studies immediately makes the value of taking a systems approach to understanding ACEng apparent. The case studies illuminate the inadequacies of reductionist approaches (case study 1), the catastrophic impact that a single widespread event (the COVID-10 pandemic) can have on ACEng (case study 2), and an example of a highly successful intervention designed with complexity in mind (the SingUp program in case study 3).

**Discussion**

 It is important to note that any framework is, to some extent, a product of reductionism; the discussion articulates the framework's literal and metaphorical nuances well but also implicitly acknowledges that capturing these nuances in visual form is challenging. The authors demonstrate critical reflexivity throughout the discussion, highlighting the subtle nuances of the theory and terminology used in the framework. Anyone applying the framework should similarly develop a working awareness of the framework's underlying theory and nuances. The discussion thoroughly covers the framework's implications for enhancing, monitoring, and researching ACEng. The limitations of the framework are thoughtfully presented.

**General comments**

 By conceptualizing the determinants of ACEng within the context of a dynamic system, the RADIANCE framework provides a radical antidote to reductive approaches to increasing ACEng. Such approaches are commonly driven by neoliberal imperatives demanding simple answers to complex issues. Case study 1, which looks at audience development initiatives in the arts, is particularly telling; consider the enormous resources (invested into shifting micro factors) that have yielded little to no return on investment! Let us hope that government and philanthropic funders take notice of the complexity captured by the RADIANCE framework and adjust their expectations accordingly.

 A *very* minor point:

 The introduction asks: what are the “determinants” of ACEng (i.e., the factors that act as facilitators or barriers to people’s engagement) and how can they be optimised? Since “determinants” are defined here as positive and negative influences on engagement, rephrasing is suggested so "optimising" doesn't refer to optimising barriers (which I'm assuming is not what the authors intended?).

Is the work clearly and accurately presented and does it cite the current literature?

Yes

If applicable, is the statistical analysis and its interpretation appropriate?

Not applicable

Are all the source data underlying the results available to ensure full reproducibility?

Yes

Is the study design appropriate and is the work technically sound?

Yes

Are the conclusions drawn adequately supported by the results?

Yes

Are sufficient details of methods and analysis provided to allow replication by others?

Yes

Reviewer Expertise:

arts in health practice and research

I confirm that I have read this submission and believe that I have an appropriate level of expertise to confirm that it is of an acceptable scientific standard.
